# Allergen-Specific Immunotherapy in Food Allergy: There Is Hope

**DOI:** 10.1097/WOX.0b013e318177ec81

**Published:** 2008-05-15

**Authors:** Johannes Ring

**Affiliations:** 1 Editor-in-Chief, Munich, Germany

## 

While the efficacy of classical subcutaneous allergen-specific immunotherapy (ASIT) has been proven by a multitude of double-blind placebo-controlled trials for various allergens and clinical conditions such as allergic rhinoconjuncitivis, allergic bronchial asthma or insect venom anaphylaxis, patients suffering from food allergy are still left with the simple recommendation of avoidance. This seems easy with regard to the major foods; a person suffering anaphylaxis to lychee can have an adequate quality of life in spite of avoidance of lychee. This is more difficult with ubiquitous food or food ingredients, which cannot be avoided so easily. Furthermore, the degree of sensitization is crucial with regard to the intensity of avoidance strategies. We recently published on the case of a woman suffering from anaphylaxis after contact with minute amounts of kiwi juice. The kiwi juice was present on a knife that had been used some time earlier to cut a kiwi. The patient herself was not even using kiwi to prepare a fruit salad; she merely used the knife with kiwi juice on it. In such cases, avoidance does not seem possible.

All this makes clear that there is a definite need for successful ASIT in food-allergic patients also. In the March posting of *WAO Journal*, we reflected briefly on the state of the art for ASIT, showing that most of the current guidelines do not recommend ASIT in food allergy; yet an increasing number of observations, case reports and pilot studies are coming up. In this issue, we read an interesting report by Wolf and Bergmann where they show that ASIT can influence the symptoms of oral allergy syndrome in pollen-associated food allergy even after sublingual application. Of course, this raises additional questions as to which route of application, which adjuvants, and which doses seem adequate when ASIT should be tried in food allergy.

The case report by Panasoff on iodide mumps shows that drug reactions can be manifold and comprise a much broader spectrum than only IgE-mediated anaphylaxis.

The study by Popov et al. illustrates the importance of sputum analyses in asthma diagnosis.

This is the 5^th ^posting of our new *WAO Journal*, the official journal of the World Allergy Organization (WAO), launched in January 2008. All members of regional and national allergy and clinical immunology societies, which are WAO members, currently have free access to this journal. Should there be some technical difficulties in logging in, please let us know, so that we can solve them. At the recent meeting of the Board of Directors of the WAO it was decided that the WAO Journal will be free for WAO members all over the world for at least one year! So please join in, read, but also write if you feel in the mood. This is a global journal and I am sure that some opinions will be controversial. In our section "Letters," we are prepared to discuss openly different points of view in controversial issues. Case reports are also welcome, when they are new or educative in nature.

The increasing prevalence of allergic diseases and the increasing complexity of our patients, both in the clinic and in the university departments, make it clear that there is a need for specialists in allergy all over the world. Two months ago we published the position statements of the WAO regarding defining the allergist and competency requirements for an allergist. Please make use of these guidelines to be active in your respective country in order to improve the training of allergy specialists worldwide!

Johannes Ring

Editor-in-Chief

Munich, Germany

